# Swallowed denture stuck in the proximal esophagus

**DOI:** 10.1007/s10354-024-01040-0

**Published:** 2024-04-18

**Authors:** Michael Habenbacher, Alexandros Andrianakis

**Affiliations:** Department of Otorhinolaryngology, Medical University of Graz, Graz, Austria

**Keywords:** Esophagus, Foreign body, Dental prothesis, Esophagoscopy, Esophageal perforation, Ösophagus, Fremdkörper, Zahnprothese, Ösophagoskopie, Ösophagusperforation

## Abstract

A 72-year-old male with dementia and Parkinson’s disease presented at the otorhinolaryngology outpatient clinic with acute dysphagia. A chest x‑ray showed a dental prosthesis in the upper esophagus, which was subsequently extracted via rigid esophagoscopy. Due to suspected esophageal perforation on postoperative CT, a cervical approach to the esophagus and flexible esophagoscopy were used, but no evidence of perforation could be identified. This case highlights challenges in managing high-risk esophageal foreign bodies in the upper esophagus, emphasizing the need for careful assessment and a multidisciplinary approach.

## Case

**Fig. 1:**
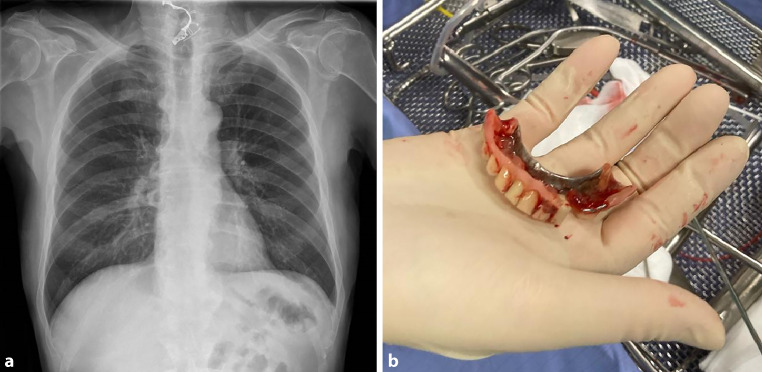
**a** Chest x‑ray showing a radiopaque foreign body shaped like a dental prosthesis in the lower cervical area. **b** Removed dental prosthesis

A 72-year-old male presented at the otorhinolaryngology outpatient clinic of a tertiary center with acute dysphagia. Severe dementia, Parkinson’s disease, and arterial hypertension were reported as the patient’s comorbidities. Pharyngeal flexible endoscopy revealed a significant accumulation of saliva in the postcricoidal area and pyriform sinuses on both sides. Upon clinical oral examination, it was noticed that the removable denture was missing from the upper jaw. Subsequent chest x‑ray showed a radiopaque foreign body resembling a dental prosthesis in the lower cervical area (Fig. 1a). A contrast swallow study was not possible due to noncompliance of the patient. The diagnosis of an ingested dental prosthesis lodged in the proximal esophagus was established.

Subsequently, rigid esophagoscopy under general anesthesia was scheduled. The denture was situated vertically in the proximal esophagus while one side of the prosthesis was located directly beneath the proximal esophageal sphincter. The mucosa surrounding the prosthesis exhibited signs of inflammation, appearing swollen and vulnerable, probably due to a prolonged impact duration. The dental prosthesis was successfully extracted in its entirety using forceps without any intraoperative objective complications (Fig. 1b), and a nasogastric tube was inserted to alleviate the vulnerable esophagus. A postoperative swallow x‑ray test indicated no signs of esophageal perforation; however, there was evidence of aspiration.

Given the escalating levels of laboratory inflammation markers during the postoperative observation period, a thoracic computed tomography (CT) was additionally performed. The imaging revealed soft tissue enlargement dorsal to the thyroid cartilage, indicative of a postoperative hematoma/edema, and a few small air inclusions in the adjacent soft tissue, raising suspicion of a perforation. Furthermore, incipient aspiration pneumonia was also diagnosed on the CT.

Due to the suspected esophageal perforation, a left lateral cervicotomy to the upper esophagus as well as a flexible esophagoscopy were subsequently performed by the thoracic surgery team; however; no evidence of macroscopic perforation could be identified during the procedures.

The postoperative course was regular without complications. After surgery, the patient was fasted and received total parenteral nutrition for support for a period of 6 days. He was then allowed to eat again perorally. Broad-spectrum antibiotic therapy was administered throughout the whole clinical course, resulting in a decline in laboratory inflammatory markers. For the rest of the antibiotic treatment, the patient was transferred to a district clinic near his home.

This report highlights the challenges in managing high-risk foreign bodies in the upper esophagus. Treatment options for esophageal foreign bodies are flexible esophagoscopy, rigid esophagoscopy, and an open cervical approach. In cases of foreign bodies situated in the upper esophagus as in the present report, rigid esophagoscopy is advised [1]. Foreign bodies lodged in the proximal esophagus pose a significantly higher risk of complications compared to those in other locations [2, 3]. A severe complication is represented by esophageal perforation, which can arise either due to the shape of the foreign body itself or come from manipulation and traction during its extraction. The risk of perforation is elevated in case of prolonged impact duration due to the induced local inflammatory process, older age, and with high-risk objects as attributed to their configuration, dimensions, and material makeup [4–6]. In our case, all aforementioned risk factors for perforation were present. In the event of suspected esophageal perforation, a contrast swallow x‑ray test is recommended to verify the diagnosis. An additional CT can provide further evidence for perforation such as enlargement of the soft tissue of the cervical mediastinum and local air inclusions. In cases of confirmed perforation, an open approach (for cervical perforations primarily a left lateral cervicotomy) is needed [1]. In the present case, the immediate postoperative contrast swallow study was negative for perforation. Due to the elevated laboratory inflammation markers, a CT of the neck and thorax was then performed. Based on the suspicion of perforation in the CT scan, a cervicotomy was performed, whereby no perforation could be detected. A further contrast study with thin barium may have ruled out a small perforation, potentially obviating the need for further surgery [1].
